# Domestic

**Published:** 1841-05-08

**Authors:** 


### DOMESTIC.

We copied in No. xvii. of the Examiner, the statistics of fractures and dislocations treated in the Pennsylvania Hospital during the last ten years. In continuation of the same subject, we extract from the New York Journal of Medicine and Surgery the following:

*Cases of Fracture in the General Hospital of Hamburgh, during the year* 1838. By Fricke.—On the 1st January, 1838, there were six cases of fracture remaining from the last year: the patients were all *men.* In the course of the year, 66 cases were received, of which 51 occurred in males, and 15 in females. The whole number treated was accordingly 72. Of these, 42 were cured, 10 died, and 20 remained under treatment.

*Ages of patients.*

| | | |
|---|---|---|
| Between 10 and 20 | - | 4 |
| " 20 " 30 | - | 16 |
| " 30 " 40 | - | 15 |
| " 40 " 50 | - | 9 |
| " 50 " 60 | - | 12 |
| " 60 " 70 | - | 10 |
| " 70 " 80 | - | 4 |
| " 80 " 90 | - | 2 |

The following table exhibits the kind of fracture, and the duration of the treatment:

| | | |
|---|---|---|
| Fracture of os nasi - - | 1 | 19 days. |
| " lower jaw - - | 1 | fatal. |
| " several ribs - | 4 | 27 days. |
| " sternum - - | 1 | fatal. |
| " clavicle - - | 3 | 31 days. |
| " os bracchii - | 7 | 95 days. |
| " radius complicated with contusion of wrist | 1 | 106 " |
| " both bones of fore-arm | 2 | 57 " |
| " bones of the pelvis | 3 | 43 " |
| " neck of os femoris | 8 | 139 " |
| " os femoris - | 9 | 94 " |
| " tibia - - - | 5 | 71 " |
| " fibula - - | 4 | 88 " |
| " both bones of leg | 22 | 96 " |
| " patella - - | 1 | 88 " |

---

*Wound of the small Intestine successfully treated by Sutures.*—M. Sullivan, aged twenty-six years, a labourer, a native of Ireland, was admitted Aug. 17th, at 4 o'clock in the morning, with a stab in the abdomen, received an hour before in a quarrel. Several knuckles of small intestine had already protruded, and his efforts to vomit were forcing out additional portions; there was also a wound of the intestine itself, three-fourths of an inch long, extending obliquely in a transverse direction, that bled freely, but was so closed by the everted mucous membrane pushing out between the edges of its peritoneal coat as to confine the air contained in the bowel and prevent it from collapsing. The protruded parts were deep red, and of natural warmth. Patient was exceedingly restless, and tossing in bed with a pale and anxious countenance; his pulse was weak and small; thirst constant, and craving cold water, of which he had already taken a large quantity, though vomiting was excited by each draught. Four sutures with fine silk were introduced into the wound of the intestine including all its tunics, and the ends cut off short. Reduction was then attempted, but was impracticable till the wound of the abdominal parietes was dilated to the extent of half an inch at its upper angle with a probe-pointed bistoury introduced upon the fore-finger of the left hand, after which it was easily effected. The external wound, which was situated to the left of the median line, midway between the umbilicus and pubes, and an inch and a half long, was closed with two sutures and adhesive straps, and over it a compress was secured by a broad bandage round the body. During the dressing he vomited several times, and soon after began to feel chilly. In four or five hours reaction came on; his pulse was eighty-eight; full and strong with continued restlessness and thirst, but no vomiting; moderate pressure on the abdomen gave pain. Ten ounces of blood were drawn from the arm, while he was supported in the sitting posture, and was followed by faintness and vomiting; after which fifty drops of laudanum were given, and iced water allowed in small quantities. At evening he was found to have been easy, and slept at intervals since the venesection. Thirst had abated, and the pulse, though rising, had not increased in frequency. Four dozen leeches were ordered to the abdomen, to be followed by large poultices of flaxseed meal, frequently renewed through the night; after the leeches, tinct. opii. gutt. L.

*Aug.* 18*th.* Patient has passed a comfortable night with some sleep; his countenance is calm; respiration natural and easy; abdomen supple and not swollen; some pain is experienced from pressure on the right side of the umbilicus; pulse is eighty-four and full, but easily compressed; temperature natural; allowed only barley water. At evening he continued easy and free from pain, but the pulse had increased in force and tension, though not more frequent. Venesection to twenty ounces was repeated, and excited sickness and vomiting, after which tinct. opii. gutt. L. were given, and the poultices continued.

19*th.* Patient has passed a good night, and is still inclined to sleep; he is free from pain, pulse is one hundred and eight, soft, and decidedly reduced in action; temperature natural; complained of weakness through the day, and vomited once without provocation. The abdomen became tympanitic and a little swollen, but without increased tenderness. He made several fruitless attempts at stool; at evening ordered a large blister to the abdomen, and an emollient enema, after which tinct. opii. gutt. xxx. It would be tedious to continue the daily history of this case; it is sufficient to state that after the 23d of August, when a second application of leeches was made over the right iliac region, the patient's convalescence was established; his bowels moving spontaneously, or by the aid of enemata, and tenderness disappearing from the abdomen, &c. The external wound healed kindly, in part by the first intention. In about five weeks he began to sit up; but was retarded afterwards by an inflammatory attack of his chest and effusion into the cavity of the peritoneum, with slight prickling pains in the lower part of the abdomen that yielded to appropriate treatment; at the time of his discharge, a slight degree of effusion still remained, though he had gained flesh and strength; when erect, a prominence of the abdomen below the umbilicus was observable with distinct fluctuation. The cicatrix left by the wound is pushed forward, and a circular opening with defined edges is felt in the parietes under the skin, for which he was advised to keep a pad and bandage applied. Discharged October 28th, 1840.—*Hospital Reports, in New York Journal of Medicine and Surgery.*

### HEALTH OF THE CITY.

INTERMENTS *in the City and Liberties of Philadelphia, from the 24th of April to the 1st of May*, 1841.

| Diseases. | Adults. | Children. | Diseases. | Adults. | Children. |
|---|---|---|---|---|---|
| Apoplexy, | 3 | 0 | *Brought forward*, | 38 | 41 |
| Cancer, | 1 | 0 | Measles, | 0 | 11 |
| Croup, | 0 | 2 | Old age, | 2 | 0 |
| Consumption of | | | Palsy, | 1 | 0 |
| the lungs, | 14 | 5 | Pleurisy, | 1 | 0 |
| Convulsions, | 0 | 6 | Scirrhus of sto- | | |
| Dropsy, head, | 0 | 1 | mach, | 1 | 0 |
| —— breast, | 2 | 0 | Small pox, | 2 | 5 |
| Disease of heart, | 2 | 0 | Still-born, | 0 | 5 |
| Drowned, | 1 | 0 | Tabes mesenteri- | | |
| Dysentery, | 1 | 0 | ca, | 0 | 1 |
| Debility, | 0 | 2 | Unknown, | 1 | 0 |
| Epilepsy, | 1 | 0 | | — | — |
| Erysipelas, | 0 | 1 | Total, | 109—46 | 63 |
| Fever, remittent, | 0 | 1 | —— | | |
| Inflammation, | 0 | 1 | Of the above, there | | |
| —— breast, | 0 | 1 | were under 1 year | | 24 |
| —— brain, | 1 | 0 | From 1 to 2 | | 18 |
| —— bronchi, | 2 | 2 | 2 to 5 | | 13 |
| —— lungs, | 0 | 15 | 5 to 10 | | 5 |
| —— stomach, | 1 | 0 | 10 to 15 | | 1 |
| —— stomach and | | | 15 to 20 | | 2 |
| bowels, | 1 | 0 | 20 to 30 | | 10 |
| —— bowels, | 3 | 0 | 30 to 40 | | 10 |
| —— liver, | 1 | 0 | 40 to 50 | | 8 |
| —— kidneys, | 0 | 1 | 50 to 60 | | 4 |
| —— heart, | 2 | 0 | 60 to 70 | | 3 |
| —— peritonæum, | 1 | 1 | 70 to 80 | | 7 |
| Intemperance, | 1 | 0 | 80 to 90 | | 4 |
| Marasmus, | 0 | 2 | | | —— |
| | — | — | Total, | | 109 |
| *Carried forward*, | 38 | 41 | | | |

Of the above there were 5 from the almshouse, and 13 people of colour, which are included in the total amount.

*Weekly Report of Interments in the City and County of New York, from the 17th to the 24th of April*, 1841.—Diseases: Apoplexy, 1; aneurism, 1; asphyxy 3; bleeding 1; burned or scalded 1; casualties 1; cancer 1; colic 1; consumption 40; convulsions 14; croup or hives 2; delirium tremens 2; dropsy 2; dropsy in the head 2; dropsy in the chest 1; drowned 2; erysipelas 1; epilepsy 1; fever, scarlet 10; do. typhoid 1; do. peurperal 1; do. remittent 1; do. bilious 1; intemprance 1; inflammation 1; do. of throat 2; do. of brain 9; do. of liver 1; do. of chest 3; do. of lungs 13; do. of bowels 3; do. of stomach 1; jaundice 2; lues venera 1; marasmus 3; measles 2; old age 1; palsy 1; spinal disease 1; sprue 1; small pox 1; unknown 3; varioloid 1.

Ages—Of 1 year and under, 30; between 1 and 2, 11; 2 and 5, 16; 5 and 10, 9; 10 and 20, 8; 20 and 30, 16; 30 and 40, 15; 40 and 50, 13; 50 and 60, 6; 60 and 70, 6; 70 and 80, 7; 80 and 90, 2; unknown 1.

